# Theory for electric dipole superconductivity with an application for bilayer excitons

**DOI:** 10.1038/srep11925

**Published:** 2015-07-08

**Authors:** Qing-Dong Jiang, Zhi-qiang Bao, Qing-Feng Sun, X. C. Xie

**Affiliations:** 1 International Center for Quantum Materials, School of Physics, Peking University, Beijing 100871, P.R. China; 2 Institute of Physics, Chinese Academy of Sciences, Beijing 100190, P.R. China; 3 Collaborative Innovation Center of Quantum Matter, Beijing 100871, P.R. China

## Abstract

Exciton superfluid is a macroscopic quantum phenomenon in which large quantities of excitons undergo the Bose-Einstein condensation. Recently, exciton superfluid has been widely studied in various bilayer systems. However, experimental measurements only provide indirect evidence for the existence of exciton superfluid. In this article, by viewing the exciton in a bilayer system as an electric dipole, we derive the London-type and Ginzburg-Landau-type equations for the electric dipole superconductors. By using these equations, we discover the Meissner-type effect and the electric dipole current Josephson effect. These effects can provide direct evidence for the formation of the exciton superfluid state in bilayer systems and pave new ways to drive an electric dipole current.

Since the idea of excitonic condensation was proposed about fifty years ago^1^,^2^,^3^, exciton systems have attracted a lot of interest. With the development of micromachining technology in the last two decades, high-quality bilayer exciton systems can be fabricated in the laboratories, in which one layer hosts electrons and the other layer hosts holes^4^,^5^. Many new interaction phenomena have been experimentally reported in the bilayer exciton systems^6^,^7^,^8^,^9^,^10^,^11^,^12^,^13^,^14^,^15^, including the vanishing Hall resistance for each layer^6^, the resonantly enhanced zero-bias inter-layer tunneling phenomenon^8^, the large bilayer counterflow conductivity^9^, the Coulomb drag^10^,^11^,^12^,^13^, etc. These phenomena strongly imply the formation of the exciton condensate superfluid state, in which many excitons crowd into the ground state. Some theoretic works have proposed several methods to detect the superfluid state^16^,^17^,^18^. However, due to the charge neutral nature of an exciton, there exists no direct experimental confirmation of the superfluid state. Thus, whether the superfluid state really forms is still unclear.

Before any further discussion, we need first to point out the specificity of excitons in bilayer systems. Because the electrons and holes are separated in space and bound with each other by the Coulomb interaction, the exciton in a bilayer system can be seen as a charge neutral electric dipole (as shown in Fig. 1a). On the other hand, superconductivity has been one of the central subjects in physics. The superconductor state has several fascinating properties, such as zero resistance^19^, the Meissner effect^20^, the Josephson effect^21^, and so on, which have many applications nowadays^22^. It is now well known that the superconductor is the condensate superfluid state of the Cooper pairs^23^, which can be viewed as electric monopoles. In other words, the superconductor state is the electric monopole condensated superfluid state. Thus, it is natural to ask whether the electric dipole superfluid state possesses many similar fascinating properties, just like its counterpart, the electric monopole superfluid state.

In this article, we will derive the London-type and Ginzburg-Landau-type equations of electric dipole superconductivity under an external electromagnetic field, and apply this theory to the bilayer exciton systems, revealing the basic characteristics of the electric dipole superconductors. Apart from the bilayer exciton systems, the electric dipole superconductor may also exist in other two or three dimensional systems, e.g., the Bose-Einstein condensate of ultracold polar molecules^24^,^25^,^26^. In fact, the ultracold polar molecules have been successfully produced in the laboratory over the past decade^24^,^25^,^26^. In addition, a new quantum state was proposed recently^27^,^28^,^29^, a magnetic dipole superconductor named the spin superconductor. Both electric and magnetic dipole superconductors contain some similar properties.

It should also be pointed out that the exciton superfluid have indeed been investigated by a lot of references in the last fifty years^1^,^2^,^3^. The Ginzburg-Landau equations of the exciton superfluid have been derived and applied extensively. Notice that the exciton’s electric dipole is usually zero, or is neglected. So there is an essential difference between the electric dipole superconductor and the exciton superfluid. In addition, a few previous works have investigated the dipole superfluid^16^,^17^,^30^,^31^. For example, Balatsky *et al.* investigated the bilayer exciton system under an in-plane magnetic field and found that the phase of the condensate can couple to the gradient of the vector potential. Therefore, the dipolar supercurrent can be tuned by the in-plane magnetic field^16^. Rademaker *et al.* predicted a quantization of magnetic flux between two layers in bilayer exciton superfluid^17^. These previous works on electric dipole superfluid only focus on a special system, namely the bilayer exciton system, and mainly investigate the effect of the in-plane magnetic field between the two layers.

Below we first derive the London-type and Ginzburg-Landau-type equations of the electric dipole superconductor. These equations can be applied to all electric dipole superconductors independent of specific systems and we apply them to study various physical properties of the electric dipole superconductor. By using these equations, we find that the Meissner-type effect and the Josephson effect of the electric dipole current. With the Meissner-type effect, an external magnetic field *gradient* can cause a super electric dipole current in an electric dipole superconductor, and a super electric dipole current can further generate a magnetic field *gradient* that is against the *gradient* of the external magnetic field. Considering the bilayer exciton systems, we show that the magnetic field induced by the super dipole current is measurable by today’s technology. We also show that the frequency in the AC Josephson effect of the electric dipole current is equal to that of the AC Josephson effect in the normal superconductor. These new effects discovered in this work can not only provide direct evidence for the existence of the exciton condensate superfluid state in the bilayer systems, but also pave new ways to drive an electric dipole current.

## Results

### London-type equations of the electric dipole superconductor

Considering a bosonic electric dipole condensate superfluid state, namely the electric dipole superconductor, under an external electric field, the force on the electric dipole **p**_0_ is


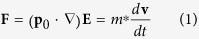


which accelerates the dipole. Here, **E** is the electric field, m^*^ is the effective mass of the dipole and **v** is its velocity. The moving electric dipole induces an electric dipole current. Due to the electric dipole **p**_0_ being a vector, the electric dipole current has to be described by a tensor 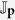. Here 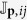 (*i*,*j* ∈ {*x*,*y*,*z*}) describes the *i*-direction current with the electric dipole pointing to the *j*-direction. In analogy with the spin current (or the magnetic dipole current)^32^,^33^, the electric dipole current can be described by 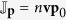 with the dipole density *n* in the classical system or 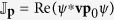 with the wave function *ψ* in the quantum system. In this work, we focus on the situation where the direction of the electric dipole **p**_0_ is fixed in the *z*-direction (as is the case in a bilayer exciton system), thus we only need a vector **J**_*p*_ = *np*_0_**v** to describe the electric dipole current with 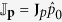, where 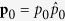 and 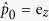 is the unit vector in the direction of **p**_0_. To combine **J**_*p*_ = *np*_0_*v* and Eq. (1), the derivative of **J**_**p**_ with respect to time is


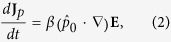


where 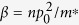. We can see that, just as **E** accelerates the super electric current^22^, 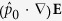 accelerates the super electric dipole current. Taking curl on both sides of the equation (2) and using the Maxwell equation^34^ ∇ × **E** = −∂**B**/∂*t*, we have 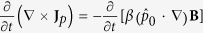. This equation suggests that we can find a conserved quantity, 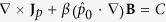 with the constant *C* being independent of the time *t*. In the present case, 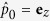 and 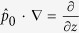. As a result, if the dipole current **J**_**p**_ = 0 deep inside the dipole superconductor, the variation of the magnetic field along the *z* direction, 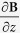, is always invariant with the time. Let us discuss the constant *C*. First, we consider a limiting case when the external magnetic field **B**^*ext*^ is zero. Then if **J**_*p*_ is zero, the magnetic field **B**^*ind*^ induced by **J**_*p*_ is also zero, the total magnetic field **B** = **B**^*ext*^ + **B**^*ind*^ = 0 and the constant *C* is zero. Obviously, in this case, the free energy of the system is the lowest and it is lower than the case with a non-zero constant *C*. Thus we take *C* to be zero due to the requirement of thermodynamic stability. Second, when the external magnetic field **B**^*ext*^ changes from zero to a finite value, the constant *C* remains zero because that it is a conserved quantity. Thus, we get the London-type equation for **J**_**p**_,


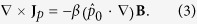


**Figure 1:**
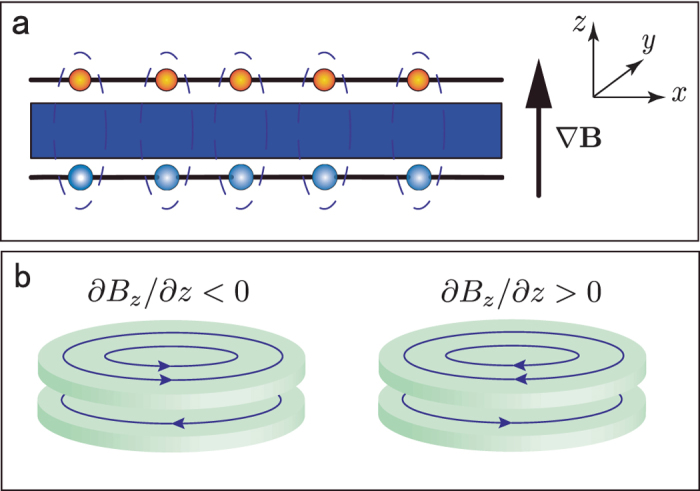
A side view of the exciton in bilayer system and the induced supercurrent by magnetic field *gradient*. (**a**) The top and bottom layers host holes and electrons respectively, and the middle blue block stands for the interlayer barrier which prevents tunneling between the two layers. (**b**) The left (right) panel shows the induced super electric dipole current for magnetic field *gradient* ∂*B*_*z*_/∂*z* < 0 (∂*B*_*z*_/∂*z* > 0). The arrows on the blue lines denote the direction of positive charge flow in each layer.

Equations (2) and (3) play similar roles as the London equations for normal superconductors^22^, so we call them the first and the second London-type equations for the electric dipole superconductor. Equation (3) implies that the *gradient* of a magnetic field **B** will induce a super electric dipole current. As is shown in Fig. 1b, if the gradient of magnetic field ∂*B*_*z*_/∂*z* < 0, the super dipole current flows in the counterclockwise direction (left panel of Fig. 1b); if ∂*B*_*z*_/∂*z* > 0, the super dipole current flows in the clockwise direction (right panel of Fig. 1b). In addition, the super dipole current can also have a feedback for an external magnetic field. The magnetic field generated by a moving electric dipole **p**_0_ with velocity **v** is equivalent to that generated by a static magnetic moment **m** = −**v** × **p**_0_^34^,^35^. As a consequence, the magnetic field induced by the super dipole current **J**_*p*_ is equivalent to that induced by the static magnetic moment distribution (magnetization) 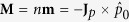. In materials, the last Maxwell equation takes the form ∇ × **B** = *μ*_0_(**j**_*f*_ + ∇ × **M** + ∂**D**/∂*t*)^34^, where *j*_*f*_ stands for the free electric current and **D** is the effective electric field. In the equilibrium case, ∂**D**/∂*t* = 0 and with no free electric current present, only the super dipole current exists, so we obtain the magnetic field equation in the electric dipole superconductor:


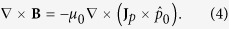


The London-type equation (3) and the magnetic field equation (4) govern the magnetic field and the super dipole current in an electric dipole superconductor. An alternative set of equations that are equivalent to equations (3) and (4) are given in Methods. From equations (3) and (4), we can obtain the Meissner-type effect against the *gradient* of an external magnetic field, which will be studied below. We can see the effect of equation (3) by considering a massless Dirac particle for which the factor *β* → ∞. In this case, the total magnetic field *gradient* ∂_*z*_*B*_*z*_ has to vanish everywhere inside an electric dipole superconductor in order to satisfy the equation (3). This means that the *gradient* ∂_*z*_*B*_*z*_ is completely screened out.

### Ginzburg-Landau-type equations of the electric dipole superconductor

Since the electric dipole condensate is a macroscopical quantum state, we can use a quasi-wave function (or the order parameter) *ψ*(**r**) to describe it. Then its free energy can be written as *F*_*s*_ = ∫_*V*_*f*_*s*_*d***r**, where *f*_*s*_ is the free energy density. In analogy with the superconductor, *f*_*s*_ can be expressed as:


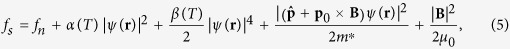


where *f*_*n*_ is the density of free energy in normal state and the momentum operator 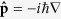. The two terms *α*(*T*)|*ψ*(**r**)|^2^ and *β*(*T*)|*ψ*(**r**)|^4^/2 are the lower order terms in the series expansion of the free energy *f*_*s*_, which have similar meanings as those in the normal superconductor^22^,^36^. Particularly, the gauge invariant term |(**p** + **p**_0_ × **B**)*ψ*(**r**)|^2^/2m^*^ can be viewed as the kinetic energy of the electric dipole superconductor (see Methods). Substitute **B** = ∇ × **A** and minimize the free energy with respect to ψ^*^ and the magnetic vector potential **A** respectively, we get (see Methods)


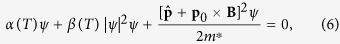



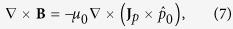


where


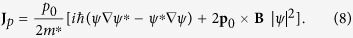


Equations (6) and (8) are the first and the second Ginzburg-Landau-type equations, respectively. They provide a full phenomenological description of the dipole superconductor. Since the velocity operator is 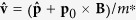, the super electric dipole current can be expressed as 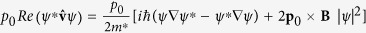. Comparing the expression 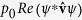 and equation (8), we find that **J**_*p*_ is exactly the super dipole current density. It should be noted that equation (7) is the same as the magnetic field equation (4). It indicates that the Ginzburg-Landau-type theory gives a more general result. Next we derive the London-type equations from the Ginzburg-Landau-type equations. The order parameter *ψ*(**r**) can be written as |*ψ*(**r**)|*e*^*iθ*(**r**)^, where |*ψ*(**r**)|^2^ is proportional to the density of dipoles *n* and *θ* represents the phase. For simplicity, we assume the amplitude |*ψ*| is the same everywhere in the dipole superconductor, whereas the phase *θ*(**r**) are allowed to change in order to account for the super dipole current. Substitute *ψ* = |*ψ*(**r**)|*e*^*iθ*(**r**)^ into equation (8), we get 
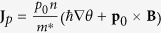
. Furthermore, if we take curl on both sides, the London-type equation (3) is recovered. It indicates that the London-type equations can be obtained from the Ginzburg-Landau-type equations, which shows the validity and consistency of our theory.

### Meissner-type effect of the electric dipole superconductor

**Figure 2:**
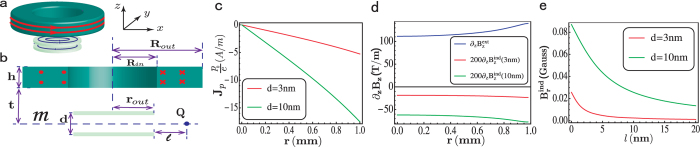
Meissner-type effect of the electric dipole superconductor. (**a**) The schematic diagram of the device consisting of a cylindrical hollow conductor and a circular bilayer exciton system (the electric dipole superconductor), and (**b**) the cross section of the device. *R*_*in*_ (*R*_*out*_) is the inner (outer) radius of the hollow conductor, and *r*_*out*_ is the radius of the dipole superconductor. *m* is the middle plane of the bilayer exciton, and *l* is distance between dipole superconductor and the point **Q** where magnetic field can be measured. *h* and *d* are, respectively, the thickness of the conductor and the dipole superconductor and *t* is the distance between them. (**c,d**) The induced super dipole current **J**_**p**_ and the gradient of the induced magnetic field 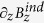 in the middle plane *m* versus radius *r*. (**e**) The induced magnetic field 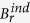 versus the distance *l*. The parameters are *R*_*in*_ = 1 *mm*, *R*_*out*_ = 1 *cm*, *h* = 1.5 *cm*, *t* = 0.1 *mm*, and *r*_*out*_ = 1 *mm*, and the thickness *d* = 13 *nm* and *d* = 10 *nm*, respectively.

In the following, we use the London-type equations to analyse the Meissner-type effect. We begin by considering a two-dimensional circular electric dipole superconductor with a radius *r*_*out*_ located in a *non-uniform* external magnetic field **B**^*ext*^ created by a cylindrical hollow conductor with an inner (outer) radius *R*_*in*_ (*R*_*out*_) and a height *h* (shown in Fig. 2a). The distance between the cylindrical hollow conductor and the dipole superconductor is *t*. Figure 2b depicts the cross-section of the device. A uniform electric current along the azimuthal direction in the hollow conductor creates a *non-uniform* magnetic field with a *gradient* 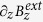 (see Supplementary), which can induce a super dipole current in the electric dipole superconductor. Substitute 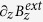 into the London-type equation (3), considering the rotational symmetry of the whole device and ∇ ⋅ **J**_*p*_ = 0, we can obtain the super electric dipole current density *J*_*p*_ at radius *r* (flowing in the azimuthal direction):


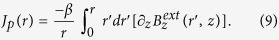


In the following, we consider that the electric dipole superconductor is the bilayer exciton system (see Fig. 1a). Then the super electric dipole current can be viewed as a counterflow electric current in the bilayer as shown in Fig. 1b and thereby induces a magnetic field **B**^*ind*^. Since the counterflow current in bilayer and the rotational symmetry about the *z* axis, the induced magnetic field **B**^*ind*^ in middle plane *m* only has the nonzero r-component 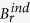. Although the z-component 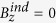, the induced *gradient* 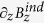 does not vanish. Figure 2d,e show 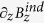 and 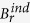 in plane *m* which can easily be calculated from the Biot-Savart law (see Supplementary).

In the calculation, we take cylindrical hollow conductor sizes as *R*_*in*_ = 1 *mm*, *R*_*out*_ = 1 *cm* and *h* = 1.5 *cm*. The current density in conductor *j* = 10^8^ *A*/*m*^2^, which generates the *non-uniform* external magnetic field **B**^*ext*^. The bilayer exciton specimen, the electric dipole superconductor, is below the conductor with *t* = 0.1 *mm* and the radius *r*_*out*_ = 1 *mm*. Here the specimen is just in the hollow region of the conductor, because 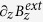 is relatively large there (see Supplementary). The two-dimensional carrier density in each layer *n* is chosen 10^12^ *cm*^−2^ and the effective mass of exciton m^*^ = 0.01 *m*_*e*_ with the electron mass *m*_*e*_. Figure 2c,d,e show respectively the induced super dipole current density *J*_*p*_, the induced magnetic field gradient 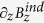 and 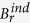 versus radius *r* for bilayer thickness *d* = 3 *nm* and *d* = 10 *nm*. A quite large *J*_*p*_ is induced near the edge of the specimen, in which the corresponding electric current density in each layer near the edge is about 15 *A*/*m*. From Fig. 2d, we find that the induced gradient 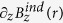 counteracts the external field gradient 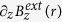. This is a Meissner-type effect in the dipole superconductor against the *gradient* of a magnetic field. Notice that it is not against the magnetic field. This is the main difference between the dipole superconductor and (monopole) superconductor. Also notice in Fig. 2d, 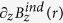 is much smaller than 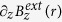 because the thickness *d* of the dipole superconductor is very small now. If for the thick dipole superconductor or for very small m^*^, 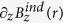 can almost be of the same value as 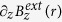, then the *gradient* of the total magnetic field vanishes inside of the dipole superconductor. Figure 2e shows the induced magnetic field 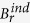 by the super dipole current, which can reach about 0.05 Gauss. This magnetic field can be accurately detected by the today’s technology. In addition, a recent work has successfully used the SQUID to detect a tiny edge current (around 0.5 *A*/*m*) in the Hall specimen^37^. In our case, the edge current density is around 10 *A*/*m*, so it should be detectable using the same method.

### The detection of the zero dipole resistance

The most remarkable phenomenon of the Bose-Einstein condensate macroscopic quantum system is superfluid, e.g. the zero resistance phenomenon of the (monopole) superconductor^19^,^22^. For the dipole superconductor, the dipole resistance is zero, i.e. the electric dipole can flow without dissipation. In the following, we suggest a method to detect the zero dipole resistance.

**Figure 3:**
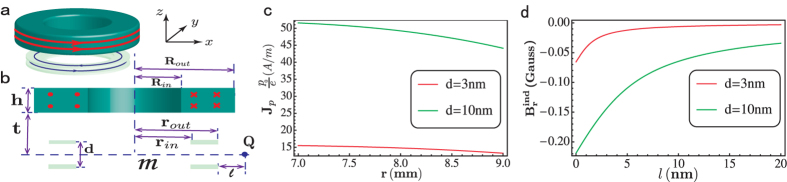
The proposed device for detection of a zero dipole resistance. (**a,b**) The schematic diagram of the proposed device and its cross section. The electric dipole superconductor is in annular shape with an inner radius *r*_*in*_ and a outer radius *r*_*out*_. Other symbols appeared in these figures have the same meanings as those in Fig. 2a,b. (c,d) The super electric dipole current and its induced magnetic field 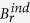 with the sizes of the dipole superconductor *r*_*in*_ = 7 *mm* and *r*_*out*_ = 9 *mm*. The other parameters are the same as in Fig. 2.

From the first London-type equation (2), we know that a variation of an electric field ∂_*z*_**E** can excite a super dipole current. This excited super dipole current will maintain for a very long time if the dipole resistance is zero. Now, consider an annular dipole superconductor specimen. This annular specimen is placed below the cylindrical hollow conductor (see Fig. 3a,b), and there 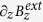 is relatively small and 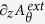 is quite large^38^ (see Supplementary). First, let the hollow conductor have an azimuthal electric current *j* and then cool the specimen into the dipole superconductor state. Next, we abruptly turn off the current *j* in the conductor. In this process, an azimuthal dipole current *J*_*p*_ will be excited. From the equation (2) and 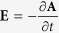, we have 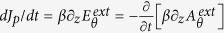. Integrating over the time *t*, we obtain the excited super dipole current:


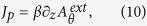


where the vector potential 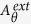 is that before the current in the conductor was turned off. Here we have used 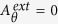 after the current turned off and *J*_*p*_ is almost zero at the beginning. Figure 3c,d show the excited super dipole current *J*_*p*_ and the magnetic field 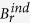 induced by *J*_*p*_. From which we find that *J*_*p*_ and 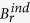 are quite large. Moreover, this excited super dipole current *J*_*p*_ does not decay for a very long time because of the zero dipole resistance. So one can measure the non-decayness of *J*_*p*_ to confirm the zero dipole resistance.

### DC and AC electric dipole current Josephson effect

The Josephson effect is another highlight of the superconductor^21^. Below we use the Ginzburg-Landau-type equations of the electric dipole superconductor to discuss the dipole current Josephson effect in a dipole superconductor-insulator-dipole superconductor junction. From the Ginzburg-Landau-type equations (6) and (8) we can see that if **B** = 0, they are the same as the Ginzburg-Landau equations of the superconductor when **A** = 0. Therefore, the DC Josephson effect of the dipole superconductor and the (monopole) superconductor are similar, *i.e.*, the super electric dipole current is *j*_*p*_ = *j*_0_ sin *γ*_0_, where *γ*_0_ = *γ*_1_ − *γ*_2_. *j*_0_ is the Josephson critical super dipole current, and *γ*_1_, *γ*_2_ are phases of the dipole superconductors.

Now we study the AC dipole current Josephson effect and consider an electric field variation ∂_*z*_*E*_*x*_. From the first Ginzburg-Landau-type equation (6), we can get the change of the phase when the super dipole current (in *x*-direction) passes through the Josephson junction. Its expression is 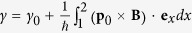. Taking the derivative with respect to time, we get 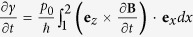. Substituting 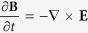, we have *γ* = *γ*_0_ − *ω*_0_*t* with 
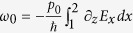
. As a result, the super dipole current can be written as *j*_*p*_ = *j*_0_ sin(*γ* − *ω*_0_*t*). It shows that the dipole current is an alternating current, although the electric field spatial variation is time-independent. We can compare this with the (monopole) superconductor. For the superconductor, a time-independent electric field (or bias) can drive an AC Josephson current. Now a spatial variation of an electric field drives an AC dipole Josephson current.

**Figure 4:**
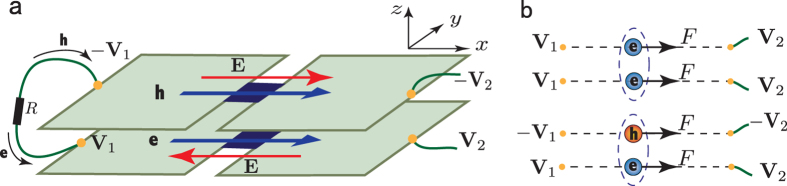
The electric dipole current Josephson junction. (**a**) The schematic diagram for the device of dipole current Josephson junction, the bilayer exciton system-insulator-bilayer exciton system junction. The left sides of the two layers are connected by a wire, and the right sides of the two layers are connected with voltages *V*_2_ and −*V*_2_, respectively. *V*_1_ and −*V*_1_ are the voltages of the left sides of the two layers. The red arrows denote the direction of the electric fields in the top layer and bottom layer, respectively, while the blue arrows represent the flowing direction of holes (electrons) in the top (bottom) layer. (**b**) The top figure and the bottom figure show the Cooper pairs (electron-electron pairs) in a ordinary superconductor under a voltage *V*_2_ − *V*_1_ and the electric dipoles (electron-hole pairs) in an electric dipole superconductor under a bilayer counter voltage, respectively. Here the Cooper pairs and the electric dipoles feel the same electric forces.

Next, we consider that the dipole superconductor is the bilayer exciton system. Figure 4a shows the schematic diagram for the device of dipole current Josephson junction. A thin wire connects the left sides of the two layers, which enables the current to flow between them. Then, if we apply the voltages −*V*_2_ and *V*_2_ to the right sides between the bilayer, it establishes an electric field in −*x* (*x*) direction in the bottom (top) layer (see Fig. 4a). This means that a spatial variation of electric field, ∂_*z*_*E*_*x*_, is added on junction, so an AC dipole Josephson current is driven and an alternating electric current emerges in the external circuit, although only a DC bias is added. For the bilayer exciton system, *p*_0_ = *ed* and ∂_*z*_*E*_*x*_ = 2*E*_*x*_/*d*, so the frequency 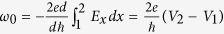. This frequency is the same with that of the AC Josephson effect of the superconductor^21^. The reason is as follows. In the electric current Josephson effect, two electrons form a Cooper pair, and the Cooper pair moves in response to external voltages. In the dipole current Josephson effect in the bilayer system, however, a pair consists of an electron and a hole, which moves in response to the counter voltages. The comparison between them is shown in Fig. 4b, in which the top figure shows the ordinary (monopole) superconductor and the bottom figure stands for the electric dipole superconductor. The only difference between them is that the voltages for the hole layer have different sign with those for the electron layer. It is clear that the electron-electron pairs feel the same electric force in the ordinary superconductor as the electron-hole pair in the dipole superconductor. Thus the frequencies of the alternating current should be the same in the two cases. The fact that the results are indeed exactly the same shows that these results of the dipole superconductor are reasonable and credible. In addition, since its frequency is the same as that of the superconductor, it is not difficult to measure in an experiment because the latter has been observed for a long time. As a result, detecting the electric dipole current Josephson effect is another feasible method to verify the formation of the dipole superconductor.

## Discussion

In conclusion, we view the exciton condensate superfluid state in bilayer electron system as an electric dipole superconductor state. Then, from the properties of the electric dipoles in an external electromagnetic field, we derive the London-type and the Ginzburg-Landau-type equations for an electric dipole superconductor. These equations are universal to all dipole superconductors and can also be used to study various properties of a dipole superconductor. By using these equations, we discover the Meissner-type effect against the *gradient* of the magnetic field and the DC and AC dipole current Josephson effects, and also suggest a method to detect the zero dipole resistance. These new effects discovered in this work can not only provide direct evidence for the existence of the exciton superfluid in bilayer electron systems, but also pave new ways to drive an electric dipole current.

## Methods

### An alternative set of equations for the electric dipole superconductor

Starting from Eqs. (3) and (4), we can get an alternative set of equations to describe the electric dipole superconductor in the steady state. First of all, make some simplification on Eq. (4), i.e,


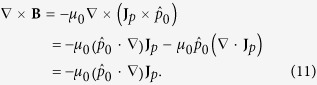


The term ∇ ⋅ **J**_*p*_ is taken to be zero in the steady state because of the conservation of the super electric dipole current in material. By taking curl on both sides of the London-type equation (3), one has 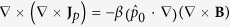. Combining this equation and Eq. (11), we obtain the equation for the super electric dipole current density


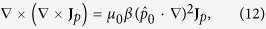


where the characteristic dimensionless ratio 
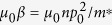
 governs the screening strength. If we take curl on both sides of Eq. (11), it transforms into 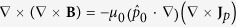. Combine this equation and the London-type equation (3), then we get the equation for magnetic field
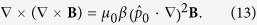


Now, we have obtained an alternative set of Eqs. (12) and (13) describing the magnetic field and the super electric dipole current separately in the electric dipole superconductor.

### The Hamiltonian of a moving electric dipole and the kinetic energy term of the electric dipole superconductor

An electric dipole moving with velocity **v** in magnetic field **B** can feel an electric field **E′** = **v** × **B**, and the corresponding energy is −**p**_0_ ⋅ **E′** = −**p**_0_ ⋅ (**v** × **B**) = **v** ⋅ (**p**_0_ × **B**)^34^,^35^. Therefore, the Lagrangian of this electric dipole is 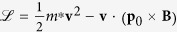, where m^*^ is the effect mass of the electric dipole. The canonical momentum is 
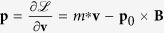
. Thus the Hamiltonian of a moving electric dipole in a magnetic field is


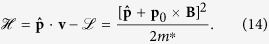


The term **p**_0_ × **B** is analogous to the term *e***A**/*c* for an electron in a magnetic field. So the kinetic energy term of the electric dipole superconductor can be written as: 
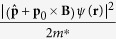
.

### The derivation of the Ginzburg-Landau-type equations of the electric dipole superconductor

First of all, we minimize the free energy shown in Eq. (5) with respect to the complex conjugate of the order parameter ψ^*^. For the second and third terms of Eq. (5), we have


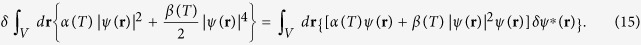


For the fourth term, we get


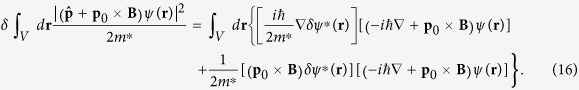


It should be noted that


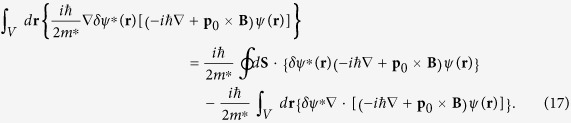


Combining Eqs. (15), (16) and (17), we can obtain:


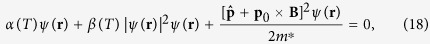


and


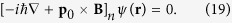


Eq. (18) is the first Ginzburg-Landau-type equation and Eq. (19) is the boundary condition for the first Ginzburg-Landau-type equation, where the subscript n stands for the component perpendicular to the surface. Here, we emphasize that this boundary condition is actually the requirement of the variational principle. In fact, if we substitute Eq. (19) into Eq. (8), we can get **J**_**P***n*_ = 0, which means that there is no electric dipole current entering or leaving the electric dipole superconductor. Similar discussions on boundary condition of the [monopole] superconductor can be found in the original paper written by Ginzburg and Landau^36^.

Next, we minimize the free energy with respect to the vector potential **A**. For the fourth term of Eq. (5), we have


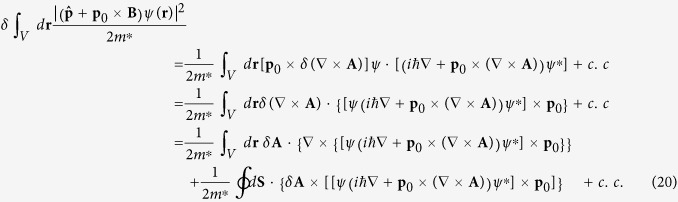


If we variate the last term of Eq. (5) with respect to vector **A**, we get


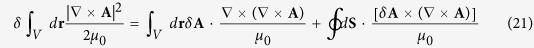


Combining Eqs. (20) and (21), we can get the second Ginzburg-Landau-type equation, i.e.,
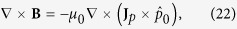
where
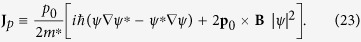


It should be noted that in the derivation the surface integral vanishes due to the requirement of free energy minimization^22^,^36^.

## Additional Information

**How to cite this article**: Jiang, Q.-D. *et al.* Theory for electric dipole superconductivity with an application for bilayer excitons. *Sci. Rep.* **5**, 11925; doi: 10.1038/srep11925 (2015).

## Supplementary Material

Supplementary Information
