# Supplementary material for: Theory for electric dipole superconductivity with an application for bilayer excitons
Source: Sci Rep. 2015 Jul 8;5:11925. doi: 10.1038/srep11925 (PMC4495569; doi:10.1038/srep11925)
Supplement: Supplementary Information [file srep11925-s1.pdf]

Supplementary Information for "Theory for electric dipole superconductivity with an application for bilayer excitons"

Qing-Dong Jiang, Zhi-qiang Bao, Qing-feng Sun, and X. C. Xie

I. The non-uniform magnetic field created by the electric current in the cylindrical hollow conductor

In the article, we consider that a non-uniform magnetic field is created by a cylindrical hollow conductor. The inner radius of the cylindrical hollow conductor is R_{in}, the outer radius is R_{out} and the length is h (see Fig. 2a and 2b in the main text). A uniform electric current density j along the azimuthal direction is applied in this conductor. In the following, we calculate the the gradient of the magnetic field caused by this electric current in the cylindrical hollow conductor. This non-uniform magnetic field is applied on the electric dipole superconductor specimen.

To deduce the magnetic field caused by the electric current in the hollow conductor, we start from the analytical expression of the magnetic field induced by a circular current loop of radius R carrying current I. The circular current loop lies in the $z = z_0$ plane, and is centered at z axis. Then the magnetic field $\mathbf{B}$ and magnetic vector potential $\mathbf{A}$ at (r, θ, z) (in cylindrical coordinate) can be immediately calculated through Biot-Savart law[1,2]. The results are

$$B_r(r,\theta,z) = \frac{\mu_0 I(z-z_0)}{2\pi\xi^2\eta r}[(r^2+R^2+(z-z_0)^2)E(k)-\xi^2K(k)], \tag{1}$$

$$B_\theta(r,\theta,z) = 0, \tag{2}$$

$$B_z(r,\theta,z) = -\frac{\mu_0}{2\pi\xi^2\eta}[(r^2-R^2+(z-z_0)^2)E(k)-\xi^2K(k)], \tag{3}$$

$$\begin{aligned}\partial_z B_z(r,\theta,z) =& \frac{\mu_0 I(z-z_0)}{2\pi\xi^4\eta^3}[(6R^2(r^2-(z-z_0)^2)\\ &-7R^4+(r^2+(z-z_0)^2)^2)E(k)\\ &-\xi^2(r^2-R^2+(z-z_0)^2)K(k)],\end{aligned} \tag{4}$$

$$\partial_z A_\theta(r,\theta,z) = -B_r(r,\theta,z). \tag{5}$$

In the above analytical expressions, $\xi = \sqrt{R^2+r^2+(z-z_0)^2-2rR}$, $\eta = \sqrt{R^2+r^2+(z-z_0)^2+2rR}$, $k = 1-\xi^2/\eta^2$, and $K(k)$ and $E(k)$ are the first kind and the second kind of the complete elliptic integral. By using the Biot-Savart law and the principle of superposition, we calculate the total external magnetic field gradient $\partial_z B_z^{ext}$ and the gradient of vector potential $\partial_z A_\theta^{ext}$. It gives

$$\begin{aligned}&\partial_z B_z^{ext} = \frac{\mu_0 j}{2\pi}\int_{-h/2}^{h/2} dz_0 \int_{R_{in}}^{R_{out}} dR\\ &\left\{\frac{z-z_0}{\xi^4\eta^3}\left\{\left[6R^2(r^2-(z-z_0)^2)-7R^4+\right.\right.\right.\\ &\left.(r^2+(z-z_0)^2)^2\right]E(k)-\xi^2[r^2-R^2\\ &\left.\left.+(z-z_0)^2]K(k)\right\}\right\}\end{aligned} \tag{6}$$

and

$$\partial_z A_\theta^{ext} = -B_r = -\frac{\mu_0 j}{2\pi}\int_{-h/2}^{h/2} dz_0 \int_{R_{in}}^{R_{out}} dR \left\{\frac{z-z_0}{\xi^2\eta r}\left[(r^2+R^2+(z-z_0)^2)E(k)-\xi^2 K(k)\right]\right\}. \tag{7}$$

Due to the rotational symmetry of the cylindrical hollow conductor, $\partial_z B_z^{ext}$ and $\partial_z A_\theta^{ext}$ are independent of the angle θ.

Fig. 1a and 1b in the Supplementary I show respectively the gradient of the magnetic field $\partial_z B_z^{ext}$ and $\partial_z A_\theta^{ext}$ versus the coordinate r in the specimen plane m. Here the cylindrical hollow conductor sizes are $R_{in} = 1mm$, $R_{out} = 1\,cm$ and $h = 1.5\,cm$. The current density in conductor $j = 10^8 A/m^2$. We can see that $\partial_z B_z^{ext}$ is quite large while $r < R_{in}$. So we suggest to put the electric dipole superconductor specimen in the hollow ($r < R_{in}$) if to investigate the Meissner-type effect of the electric dipole superconductor. In addition, Fig. 1a and 1b in the Supplementary I also show that the magnetic field gradient $\partial_z B_z^{ext}$ is relatively small but $\partial_z A_\theta^{ext}$ is large when below the cylindrical hollow conductor with $7mm < r < 9mm$ (see the yellow color shadow region), and that is why we choose to put the annular dipole superconductor specimen in there for the investigation of the zero electric dipole resistance.

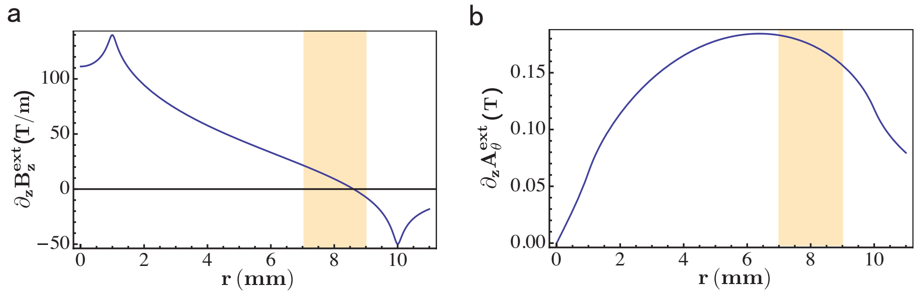

Supplementary Figure 1: **The gradient of the magnetic field and the vector potential created by the cylindrical hollow conductor.** (**a**) The gradient of the magnetic field $\partial_z B_z^{ext}$ created by the cylindrical hollow conductor versus the coordinate r in the specimen plane m. (**b**) $\partial_z A_\theta^{ext}$ versus the coordinate r in the specimen plane m. This magnetic field is applied on the electric dipole superconductor specimen. The parameters are same as in Fig. 2 in the main text.

II. The magnetic field induced by the super electric dipole current

In this section, we calculate the magnetic field induced by the super electric dipole current in the bilayer exciton system. In the bilayer exciton system, the electric dipole current can be viewed as counter-flow electric currents. Since the bilayer system has the reflection symmetry about the middle plane m and the rotational symmetry about the z axis, the induced magnetic field in plane m only has the nonzero r-component B_r. Although the z-component of the induced magnetic field vanishes in plane m, the magnetic field gradient $\partial_z B_z^{ind}$ does not vanish. Moreover, we note that the induced magnetic field B_r^{ind} and its gradient $\partial_z B_z^{ind}$ are twice of that induced by the electric current in one layer. We will calculate the magnetic field induced by the electric current in one layer, and then times 2. At last, we obtain $\partial_z B_z^{ind}$ and B_r^{ind} in plane m:

$$\partial_z B_z^{ind}(r) = \frac{\mu_0}{2\pi}\int_0^{r_{out}} dR \left\{\frac{J_p}{\xi_0^4\eta_0^3}[(6R^2(r^2-(d/2)^2)-7R^4+(r^2+(d/2)^2)^2)E(k_0)-\xi_0^2(r^2-R^2+(d/2)^2)K(k_0)]\right\} \tag{8}$$

$$B_r^{ind}(r) = -\frac{\mu_0}{2\pi}\int_0^{r_{out}} dR\left\{\frac{J_p}{\xi_0^2\eta_0 r}[(r^2+R^2+(d/2)^2)E(k_0)-\xi_0^2K(k_0)]\right\} \tag{9}$$

where $\xi_0 = \sqrt{R^2+r^2+(d/2)^2-2rR}$, $\eta_0 = \sqrt{R^2+r^2+(d/2)^2+2rR}$ and $k_0 = 1-\xi_0^2/\eta_0^2$.

Supplementary References

[1] Griffiths D. J. *Introduction to Electrodynamics 4th ed.* (Pearson, London, 2013).
[2] Simpsons, J., Lane, J., Immer, C. & Youngquist, R. Simple Analytic Expressions for the Mangetic Field of a Circular Current Loop. *Tech. Rep. NASA*, (2011).
